# Virtual Sensing and Virtual Reality: How New Technologies Can Boost Research on Crowd Dynamics

**DOI:** 10.3389/frobt.2018.00082

**Published:** 2018-07-13

**Authors:** Mehdi Moussaïd, Victor R. Schinazi, Mubbasir Kapadia, Tyler Thrash

**Affiliations:** ^1^Center for Adaptive Rationality, Max Planck Institute for Human Development, Berlin, Germany; ^2^Chair of Cognitive Science, Department of Humanities, Social, and Political Sciences, ETH Zurich, Zurich, Switzerland; ^3^Computer Science, Rutgers University, The State University of New Jersey, New Brunswick, NJ, United States; ^4^Geographic Information Visualization and Analysis, Department of Geography, University of Zurich, Zurich, Switzerland; ^5^Digital Society Initiative, University of Zurich, Zurich, Switzerland

**Keywords:** pedestrians, collective movement, complex systems, social interactions, tracking, virtual environment

## Abstract

The collective behavior of human crowds often exhibits surprisingly regular patterns of movement. These patterns stem from social interactions between pedestrians such as when individuals imitate others, follow their neighbors, avoid collisions with other pedestrians, or push each other. While some of these patterns are beneficial and promote efficient collective motion, others can seriously disrupt the flow, ultimately leading to deadly crowd disasters. Understanding the dynamics of crowd movements can help urban planners manage crowd safety in dense urban areas and develop an understanding of dynamic social systems. However, the study of crowd behavior has been hindered by technical and methodological challenges. Laboratory experiments involving large crowds can be difficult to organize, and quantitative field data collected from surveillance cameras are difficult to evaluate. Nevertheless, crowd research has undergone important developments in the past few years that have led to numerous research opportunities. For example, the development of crowd monitoring based on the virtual signals emitted by pedestrians' smartphones has changed the way researchers collect and analyze live field data. In addition, the use of virtual reality, and multi-user platforms in particular, have paved the way for new types of experiments. In this review, we describe these methodological developments in detail and discuss how these novel technologies can be used to deepen our understanding of crowd behavior.

## Introduction

Understanding crowd movementsis key to the management of dense pedestrian flows in urban areas. Research on crowd dynamics can inform urban planners and help authorities design efficient public places in order to avoid congestions and enhance traffic efficiency (Cassol et al., 2017; Haworth et al., 2017). In addition, crowd research can save lives in extreme situations (Helbing et al., 2014). Recent studies have shown that the frequency and severity of deadly crowd accidents have increased over the past decades (Helbing et al., 2007, 2014; Helbing and Mukerji, 2012). In September 2015, one of the most dramatic crowd stampedes occurred in Mecca during which thousands of pilgrims were crushed to death in a dense crowd (Khan and Noji, 2016). This tragedy is one example of a series of accidents that have occurred in the past decade, costing many lives and undermining trust in public institutions. In the present article, we will describe new technologies that can potentially transform the way crowd researchers address these fundamental issues.

### How the system works

Pedestrian crowds belong to a large family of self-organized social systems (Helbing et al., 2005; Moussaïd et al., 2009), including animal swarms (Camazine, 2003) and human activities such as judgment formation and consumer behaviors (Castellano et al., 2009; Moussaïd et al., 2015). In such systems, the collective dynamics of the group is driven by behavioral propagation processes that are induced by interactions between individuals (Moussaïd et al., 2017). Indeed, pedestrian behaviors tend to spread from person to person, resulting in large-scale snowball effects. For example, when pedestrians slow down or stop in the middle of a dense crowd, they force followers to also slow down or stop in order to avoid a collision. This can trigger a chain reaction as others adapt their movement and/or speed. Behaviors as diverse as choosing an exit door, avoiding others on a particular side, pushing, or escaping from danger are subject to behavioral propagation. This propagation process eventually gives rise to collective patterns, such as lane formation, the emergence of trail networks, and biases in exit choice (Helbing et al., 2005). For example, crowd turbulence is a deadly collective phenomenon that has been recently identified from video surveillance analyses and systematically associated with crowd accidents (Helbing et al., 2007). This pattern is characterized by the occurrence of waves of pushing that propagate from person to person through the crowd. At very high densities, body contacts between neighboring individuals support the spread of pushing forces. These pushing waves set up, merge, and amplify when a certain density threshold is achieved. As a result, people can be trampled by others or crushed against walls. Thus, a large-scale global pattern (e.g., crowd turbulence) can emerge from a simple propagative individual behaviors (e.g., pushing behaviors).

The link between global patterns and the individual behaviors that cause them is often difficult to establish. A crowd is more than a collection of many isolated individuals. Studying individual behaviors in isolation is not sufficient for understanding collective dynamics, and macroscopic descriptions of these patterns are not informative regarding the mechanisms underlying their emergence. Instead, one needs to focus on the causal mechanisms underlying these two levels of observation (i.e., individual and collective behaviors).

### How to study the crowd

In order to study crowd behavior, researchers use a combination of computer simulations, field observations, and laboratory experiments. Computer simulations explore the conditions in which collective behaviors can emerge by simulating the movements and interactions of many individuals. The outcomes of simulations are determined by behavioral models that describe how individuals respond to their physical and social environments. Existing microscopic pedestrian models include behavioral elements such as how individuals walk to their destinations, how they avoid obstacles, and how they adapt to the presence of other individuals. A large variety of models have been developed in the past. These models include physics-based models (Helbing and Molnár, 1995), biomechanically-based approaches (Singh et al., 2011b), vision-based models (Ondrej et al., 2010; Moussaïd et al., 2011; Dutra et al., 2017), velocity-based approaches (Guy et al., 2009; van den Berg et al., 2011), and hybrid approaches (Singh et al., 2011a). In addition, macroscopic models aim at describing crowd movement by means of locally averaged quantities, such as the velocity, density, or flow of individuals. This type of model is often inspired by Henderson's original specification with respect to fluid dynamics (Henderson, 1974). The state-of-the-art for crowd modeling techniques has been reviewed in several articles (e.g., Bellomo and Dogbe, 2011; Schadschneider et al., 2011; Degond et al., 2013) and is beyond the scope of this article. A key challenge is to capture the essence of real human crowd behavior while generalizing to future scenarios (e.g., a change in environmental conditions or stress induction in a crowd).

Another methodological approach consists of collecting real-world data directly in the field (e.g., Gallup et al., 2012; Alnabulsi and Drury, 2014). These empirical observations can be used to build data-driven computational models of human crowds (Qiao et al., 2017). Researchers typically set up video recording installations directed at crowded urban environments or use existing recordings from video surveillance platforms. The recorded walking behaviors of pedestrians can then be quantified by reconstructing the positions of individuals from the video images. The advantages of studying real-world phenomena are often undermined by difficulties with the accuracy of these reconstructions, particularly for dense crowds. This quantification step is usually undertaken by means of computer vision software (e.g., Pérez-Escudero et al., 2014) but often requires the tedious efforts of research assistants.

The third approach to studying crowd behavior is to conduct controlled laboratory experiments. In a typical experiment, researchers will invite a group of participants to the laboratory and provide them with specific walking instructions. In the past two decades, a large number of experiments have involved up to hundreds of participants simultaneously, covering a wide range of scenarios. These experiments investigated the study of crowd evacuations, density effects, patterns characterizing uni- and bi-directional flows of people, and large-scale evacuations from public buildings (Hoogendoorn and Daamen, 2005; Jelić et al., 2012; Moussaïd et al., 2012; Burghardt et al., 2013; Wagoum et al., 2017). The popularity of crowd experiments can be explained by the potential to vary experimental factors in a controlled manner, coupled with the ease of tracking participants positions with dedicated tracking devices.

### New perspectives

New technologies such as virtual sensing and multi-user virtual reality platforms can complement the opportunities afforded by field observations and laboratory experiments. Virtual sensing consists of estimating crowd movements by tracking the Wi-Fi and Bluetooth signals emitted by pedestrians' smartphones. Whereas, the idea of estimating a quantity by means of a proxy measure is typically found in other domains (e.g., computer science, chemistry, or transportation science; Liu et al., 2009), this methodology also constitutes a promising line of research for crowd monitoring. In addition, the emergence of multi-user virtual reality platforms can be used to study the movement behavior of crowds instead of individual participants. Controlled crowd experiments have recently been conducted in virtual environments, extending the limits of possible experimental designs (Thrash et al., 2015; Moussaïd et al., 2016).

We describe how the emergence of virtual sensing and virtual reality can boost crowd research, their potential applications, and corresponding challenges. In the following section, we present previous crowd monitoring techniques and the potential of smartphone-based signals. This section is followed by a discussion of virtual reality from single-user experiments to recent development in multi-user virtual environments. The article concludes with a discussion that highlights the future promises of these techniques for field observations and controlled experiments.

## Crowd monitoring in the field

Crowd monitoring involves collecting quantitative information about an existing crowd located in an area of interest, such as crowded streets, music festivals, or train stations. Unlike laboratory experiments and computer simulations, crowd monitoring provides data on real-world behaviors with high external validity. The obtained data may include (i) macroscopic features of the crowd (e.g., density, flow, movement patterns) and/or (ii) microscopic information regarding the pedestrians (e.g., their positions in space, walking trajectories, walking speeds). However, accurate monitoring can be challenging in practice. Crowd monitoring often requires tedious manual corrections and tailored adjustments to specific external factors (e.g., calibrating video analyses techniques to ambient light conditions). There are at least two categories of technical options for monitoring crowds (i.e., conventional methods and virtual sensing).

### Conventional methods

Conventional methods of crowd monitoring include manual crowd counting and computer vision. An early procedure for manual crowd counting was introduced by Herbert Jacobs in 1967—a journalism lecturer at the University of California at Berkeley (Jacobs, 1967). During the Berkeley riots against the Vietnam war, Jacobs observed a crowd from his office window and devised what is known as the “Jacobs method” for estimating its size. The Jacobs method involves estimating the number of people within a square of a stone pavement grid and counting how many of these squares were occupied. Crowd density can then be estimated by calculating the number of people per square meter. This method is still frequently used to estimate crowd density based on video surveillance footage (Raybould et al., 2000). To date, the Jacobs method also remains a simple procedure for extracting the ground truth values used as benchmarks in the validation of more sophisticated methods. Other manual counting approaches include counting people with digital clickers at entrance or exit gates (Bauer et al., 2009, 2011).

Given recent advancements in technology, computer vision techniques have become increasingly popular. This technique consists of extracting relevant crowd information based on the automated analyses of videos. These videos are often sourced from surveillance cameras or aerial images. There are two distinct approaches to computer vision, including the direct approach of detecting people's bodies (Rittscher et al., 2005) or faces (Lin et al., 2001) and the indirect approach of inferring the presence of people using image transformation procedures. For example, researchers have used indirect methods by counting foreground pixels after subtracting the background image (Davies et al., 1995; Ma et al., 2004). Other researchers have employed texture features analysis (Marana et al., 2005), histograms of edge orientations (Dalal and Triggs, 2005), and moving corner points to estimate the number of moving people (Albiol et al., 2009). Crowd flow may also be estimated using the frame difference algorithm (Liang et al., 2014) or the optical flow approach (Andrade et al., 2006).

In the recent years, computer vision techniques have been reshaped by the rise of deep learning (Ouyang and Wang, 2013). Convolutional neural networks can be trained on large hand-annotated crowd datasets (e.g., ImageNet, WWW crowd dataset) to associate image features with higher-level information about the crowd. These methods can produce microscopic quantities, such as the position, number, and trajectories of the pedestrians (Ouyang and Wang, 2012, 2013; Sermanet et al., 2013), or macroscopic information, such as density maps (Sindagi and Patel, 2017), the spatial distribution of the crowd (Kang and Wang, 2014), and contextual information regarding what kind of crowd is present, where the scene occurs, and reasons for the gathering (Shao et al., 2015, 2017). Because deep learning can handle common problems that hinder the efficiency of traditional approaches (e.g., changing camera perspective, body occlusions, and lighting conditions), accuracy levels are typically higher than what can be achieved by conventional methods (Tian et al., 2015).

Despite the fast development of deep learning and the attention it has received in the domain of computer science, this method has not yet widely reached the community of crowd researchers. This is probably related to its lower accessibility for non-experts and the technical complexity of its implementation. To date, traditional crowd monitoring methods remain relatively popular, but the promises of deep learning foreshadow an important development in the near future.

### Virtual sensing

Whereas, conventional methods aim to visually detect the presence of people (with the human eye or the computer eye), virtual sensing consists of detecting traces of people and inferring their numbers, density, and movements. Many methods of crowd sensing rely on emerging technologies that enable the detection of physical and virtual traces left by pedestrians. These methods include carbon dioxide sensors (Ang et al., 2016), audio sensors (Kannan et al., 2012), floor pressure sensors (Mori et al., 2004), seismic sensors (Damarla et al., 2016), motion sensors (Coşkun et al., 2015), and radar sensors (Choi et al., 2016).

In our highly connected world, people do not only leave physical traces in their environment but also emit a variety of virtual traces (e.g., the radio-frequency signals produced by smartphones or other electronic equipments). The increased reliance on smartphones and other connected devices has motivated researchers to extract the crowd information provided by these mobile devices (Eagle et al., 2009; Ding et al., 2015). Numerous applications have been developed that employ smartphones as sensors for the recognition of activities such as mobility, health information, and social interactions (see survey in Khan et al., 2013). In the specific case of crowd sensing, collecting location data from smartphones can be achieved by accessing a device's GPS or Wi-Fi positioning information (with positional accuracies of ~5 and 20 m, respectively; Azizyan et al., 2009; Van Diggelen, 2009).

However, collecting position information is not trivial. For privacy reasons, the positioning information of any randomly selected pedestrian is typically not publicly available. Some researchers have circumvented this challenge by setting up a voluntary participatory system. Here, volunteers can register to participate in the study and install an experimental application on their smartphone. The application continuously records the user's spatial position and sends it to a central server. Recent studies have shown that many individuals are willing to install such an application and share this type of data as long as the scientific use of this data is communicated in a transparent manner and when the participants can receive valuable information in return (Wirz et al., 2012). For example, participants recruited at a music festival may be able to use the application to access an interactive program guide, a map of the neighboring points of interest, background information regarding ongoing concerts, and other social features. Furthermore, the application can be used to send personalized location-dependent information to the users. For example, the police can inform attendees located in a particular area about how to behave in case of an emergency. One challenge of this sensing method is that the researcher cannot expect to receive position information from all individuals in the area of interest. Because only a fraction of people will be using the application, researchers must extrapolate the positions and movements of the entire crowd from those of the collected sample.

**Figure 1:**
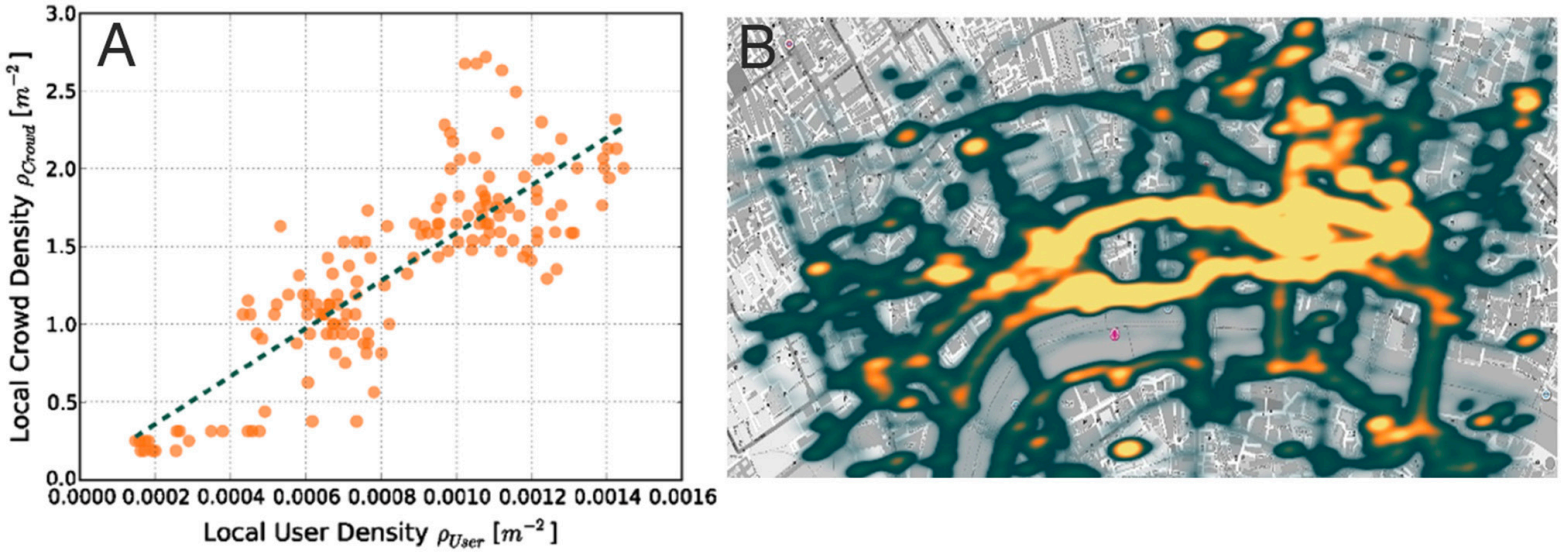
Virtual sensing of recruited participants **(A)** Correlation between the application user density and the actual crowd density **(B)** Map indicating the estimated crowd density during the 2011 Lord Mayor Show in London (from Wirz et al., 2013).

This method has been previously employed during the 2011 Lord Mayor Show in London (Wirz et al., 2013) and the 2013 Züri Fäscht in Zürich (Blanke et al., 2014). During the Lord Mayor Show, 828 users downloaded the application (out of nearly half a million visitors) and ~4 millions GPS positions were collected at a sampling rate of 1 Hz. This method was validated by comparing the GPS position data to a ground truth sample resulting from the semi-automatic monitoring of surveillance camera recordings. This study demonstrated that the application users were distributed across the festival area similarly to the rest of the crowd. Indeed, there was a positive correlation between the density of application users and the actual crowd density (Figure 1A). As an illustrative result, Figure 1B shows a map of the crowd density in the festival area.

For the 2013 Züri Fäscht—a 3-day event comprising concerts and shows—the scaling was considerably increased. Out of 2 million total visitors, 28,000 users downloaded the application, resulting in ~25 million location updates. The higher participation rate of this second deployment resulted from an important marketing effort in promoting and distributing the application. Several functionalities were added, including a “friend finder” that allowed users to locate their friends in the event they became lost in the crowd. The gamification of this application (with a “trophy collector” function) also possibly contributed to the higher download rate. Finally, a link to the user's Facebook profile favored the viral propagation of the application on social networks.

Overall, this application allowed for the collection of detailed data regarding the crowd at a scale and with an accuracy that was rarely achieved in the past.

Despite the advantages of virtual sensing with active participants, this method relies on an intensive marketing effort. Alternatively, researchers may track pedestrians passively using the Bluetooth and Wi-Fi signals emitted by their mobile devices. Indeed, Bluetooth and Wi-Fi signals can be detected using dedicated scanners (Musa and Eriksson, 2012; Barbera et al., 2013). When applied to crowd observation, stationary scanners positioned in the area of interest can allow the detection of virtual traces left by pedestrians and thus the estimation of their number and displacement (Fukuzaki et al., 2014; Schauer et al., 2014). Hence, pedestrians do not need to actively cooperate with the researchers by downloading an application on their phones.

However, the deployment of the scanners can be challenging. One important issue with Wi-Fi is the interruption of the signal propagation path caused by solid obstacles located between the source and the scanners. In addition, human bodies can also produce a shield effect that causes fluctuations in the signal. One solution is to mount them *above* the crowd, thus enabling a free line of sight toward the devices. While this solution is easily applicable in indoor environments, it is more challenging when tracking people in open spaces, such as commercial walkways or music festivals.

Virtual sensing with passive participants has been successfully deployed several times in the past (e.g., in shopping malls, car exhibitions, and airports, see Fukuzaki et al., 2014; Schauer et al., 2014). For example, Weppner et al. (2016) used a setup consisting of 31 scanners (covering a total area of ~6,000 m^2^) during the IAA car exhibition in Frankfurt. Data was collected for 13 business days, producing nearly 90 million data points from a total of over 300,000 unique mobile devices. A video-based manual counting procedure was also employed in order to validate the virtual sensing data. The scanners were mounted on the ceiling with an average distance of 14 m between them and an average scanning zone of 180 m^2^ for each of them.

**Figure 2:**
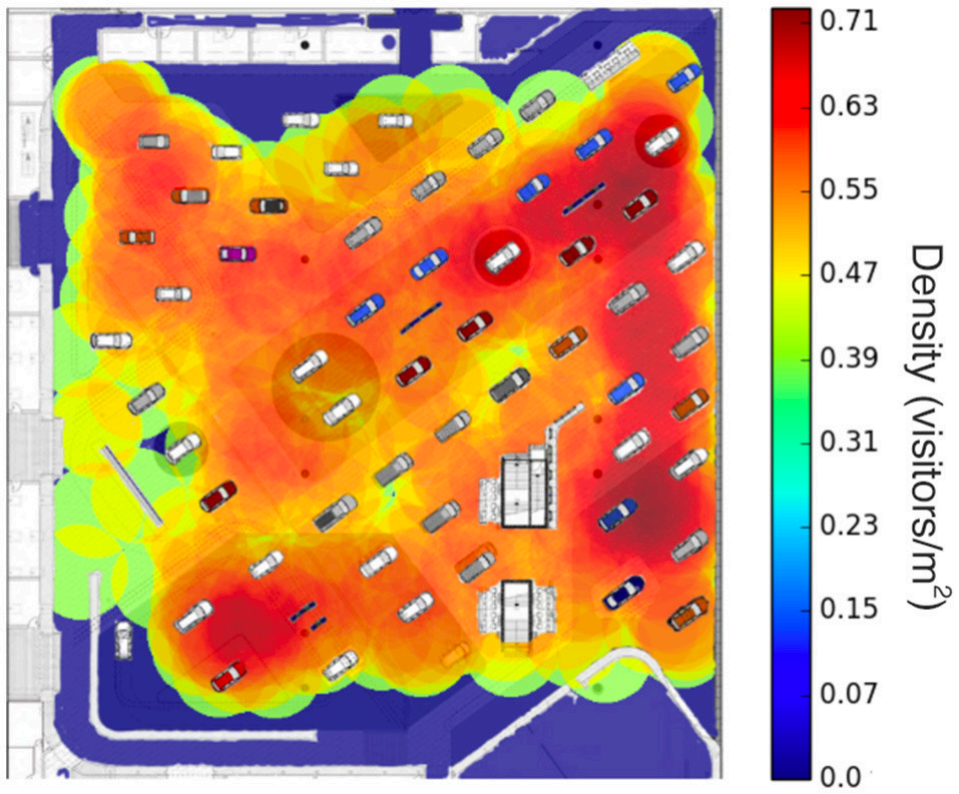
Virtual sensing of passive participants. This map shows the estimated crowd density during the Frankfurt car exhibition (from Weppner et al., 2016).

Whenever, pedestrians walked through the detection area, the Bluetooth and Wi-Fi signals emitted by their mobile devices were detected by the scanners and sent to a central database server. Every incoming signal was associated to an RSSI value (i.e., the Received Signal Strength Indication). This information can be combined with the coordinates of the scanners to estimate the location of the pedestrian during a post-processing phase. Multiple scanners can detect the presence of a unique mobile device at a given moment of time. The simplest localization method is to assign the spatial coordinates of the scanner that has recorded the highest RSSI (i.e., the strongest signal) to the pedestrian. A more sophisticated method is based on an RSSI-weighted average of the scanners locations. In a preliminary accuracy evaluation phase, the positioning error was estimated to a maximum of 10 m for 90% of the devices. Figure 2 shows the estimation of local densities in subregions delimited by the boundaries of a Voronoi cell surrounding each scanner.

Calibration was necessary to convert the estimated density of people into the actual density because not all visitors were carrying a detectable device and the signal was not always detected. Toward this end, ground truth manual measurements were compared to the measures provided by the sensors. Weppner et al. (2016) calculated that the measures provided by the sensors have to be multiplied by an average of 1.5 in order to match the ground truth values. In practice, the value of the multiplier might vary depending on social and environmental conditions and would need to be calibrated by means of preliminary evaluation data.

## Virtual reality in the laboratory

Virtual reality (VR) is a technology that involves presenting a person with a responsive artificial environment. Participants in VR studies are typically able to look around, move in, and interact with the virtual environment. As such, VR constitutes an interesting opportunity to study pedestrians' behaviors such as locomotion (i.e., bodily movement through the immediate environment) and wayfinding (i.e., spatial decision-making in a large-scale environment; Montello, 2005).

### Techniques and single-user experiments

In VR, the interaction between a navigator and the environment is mediated by a display (e.g., projection screen, head-mounted display), and a control interface (e.g., a joystick, a mouse, and keyboard, head movement sensors). Large projection screens and desktop displays often provide a more natural field of view but do not always allow users to rotate their bodies 360° in order to experience the virtual environment (but see Höllerer et al., 2007). In contrast, head-mounted displays (HMDs) are relatively mobile and restrict visual access to the external world (e.g., Oculus Rift, https://www.oculus.com/; HTC Vive, https://www.vive.com/us/) (see e.g., Chance et al., 1998; Waller et al., 2004; Foo et al., 2005; Kinateder and Warren, 2016). One consequence of using VR displays is that distances are systematically underestimated to a greater extent than distances estimated in the real world (Knapp, 2003). However, training in VR that involves explicit visual feedback can reduce these biases (Richardson and Waller, 2005). Similarly, spatial updating has been found to be less precise in VR without physical turns (Klatzky et al., 1998), but biases in turn perception *per se* can be reduced with explicit visual feedback (Bakker et al., 2001).

The control interface translates the movements of users into visual feedback on the display. Two important aspects of control interfaces are the position of the body (Taube et al., 2013) and the possible ways in which specific actions (e.g., pushing a joystick forward) are connected with specific types of feedback (e.g., the expansion of optic flow). During locomotion in VR, the user's body can be sitting (e.g., Richardson et al., 1999), lying (as in neuroscientific research; Taube et al., 2013), or standing (e.g., Nescher et al., 2014). While sitting or lying (or standing in place), the user does not receive proprioceptive (i.e., body-based) feedback. In addition, lying causes a conflict in perspective between facing upwards in the real environment (e.g., the fMRI scanner) and facing forward in the virtual environment (Taube et al., 2013). Comparisons of control interfaces are often case-specific. For example, Thrash et al. (2015) found that users' performance on navigation-related tasks was more efficient and less error prone with a mouse-and-keyboard setup than a handheld joystick. However, less attention has been allocated to theoretical explanations for why users tend to perform better with some interfaces than others. While mouse-and-keyboard setups are often more familiar than joysticks, the extent to which one interface is more “intuitive” than the other is unknown (Lapointe et al., 2011). This challenge may be addressed in the future by studies that focus on the impact of training on interface use or on how to allow for realistic walking in VR.

For realistic walking, some researchers have employed omnidirectional treadmills (as a hardware solution; e.g., Souman et al., 2010) and redirected walking algorithms (as a software solution; e.g., Razzaque et al., 2001). Redirected walking steers users toward particular targets by expanding and compressing rotations and translations and allows for locomotion through environments that are larger than the external infrastructure. Even when VR participants walk with an HMD (without these visual distortions), the HMD necessarily translates head movements into visual feedback and thus constitutes a control interface.

Advancements in control interface technology will be critical for studies of locomotion but may be less critical for studying certain aspects of wayfinding. Indeed, during wayfinding, the decisions executed by the navigator typically depend less on physical collisions or maneuverability than incomplete mental representations and salient environmental cues.

Wayfinding behavior can be classified as either path integration or landmark-based (Taube et al., 2013). During path integration, observers rely on idiothetic cues in order to maintain their orientations and positions during movement through a large-scale environment (Gallistel, 1990). Landmark-based navigation relies primarily on allothetic cues (e.g., visible objects along a route; Presson and Montello, 1988) and is associated with scene processing (Epstein and Vass, 2014) and survey representation (Kitchin and Blades, 2002). Indeed, this type of wayfinding has been successfully studied using a variety of VR systems, including projection screens in fMRI scanners (Epstein et al., 2017), desktop displays with simple controls (Waller and Lippa, 2007), and HMDs with naturalistic walking (Hodgson et al., 2011).

Virtual reality has allowed real humans to interact with their digital counterparts (i.e., avatars) in an effort to study more detailed local interactions under controlled experimental conditions. For example, Olivier and colleagues have used VR in order to study how people avoid collisions with groups (Bruneau et al., 2015), the impact of social roles on collision-avoidance strategies (Olivier et al., 2013), as well as human-robot interactions (Vassallo et al., 2017). Similarly, Warren and colleagues have focused on human locomotion and spatial navigation using VR (Bonneaud et al., 2012). These studies have allowed researchers to test theories of perceptual-motor control and develop a formal model of pedestrian behavior (Warren and Fajen, 2004; Bonneaud and Warren, 2012). This model has been expanded to include perception (Bruggeman et al., 2007; Warren and Fajen, 2008) and behaviors such as target interception (Fajen and Warren, 2007) and collision avoidance with both static and moving objects (Fink et al., 2007).

### Immersive multi-user experiments

One drawback of single-user experiments is the lack of interactions between participants. The collective dynamics of a crowd cannot be explained by the accumulation of many isolated individuals. Rather, collective behaviors stem from social interactions between pedestrians. Observing the interactions of a single participant with simulated agents constitutes an interesting step toward studying crowd dynamics in VR (Drury et al., 2009). Nevertheless, insight into collective behavior remains elusive because the dynamics of the group are largely determined by the behavior of the virtual agents implemented by the experimenter.

This challenge has been recently addressed with the development of *multi-user* virtual environments (Normoyle et al., 2012; Bode and Codling, 2013; Bode et al., 2014; Carlson et al., 2014; Moussaïd et al., 2016; Boos et al., 2017). These multi-user environments enable the observation of a *crowd* of participants moving and interacting in a shared virtual environment simultaneously. In a typical multi-user experiment, every participant controls an avatar in the virtual environment from a first-person perspective. The avatars can view and interact with each other in real time (e.g., avoiding, following, or colliding) and thus mimic some aspects of social interactions among real pedestrians. In the following series of experiments, Moussaïd et al. (2016) explored the potential of multi-user VR using desktop displays with a mouse-and-keyboard control interface (Thrash et al., 2015).

### Validation

Given the novelty of multi-user VR experiments, initial research has focused on validating simple crowd behaviors observed in virtual worlds. Here, we describe two studies that have compared avoidance maneuvers and simple evacuation situations against real-world data.

#### Side preference

**Figure 3:**
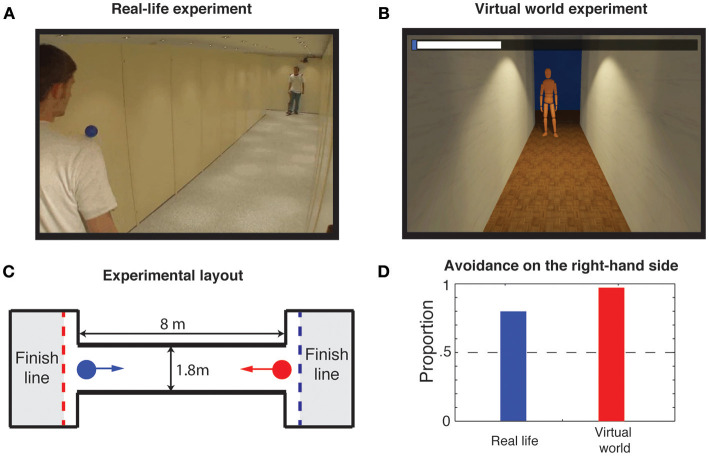
The side preference. **(A)** Illustration of the real-life experiment in which pairs of participants were instructed to avoid each other in a narrow corridor (Moussaïd et al., 2009). **(B)** Replication of the side preference experiment in the multi-user virtual environment. **(C)** The layout and the dimensions of the corridor are identical between the two experiments. **(D)** In both experiments, participants exhibited a marked preference for avoidance on the right-hand side (81% in the real-life, 95% in the virtual environment), demonstrating that people rely on the same social norms in real and virtual settings.

Avoidance maneuvers between pedestrians are characterized by a well-known social bias called the side preference (Helbing, 1992). In most Western countries, people preferentially evade each other on the right-hand side. This bias is a *social* attribute that does not occur during the avoidance of a static obstacle (Moussaïd et al., 2009). In a multi-user VR experiment, 95% of the participants exhibited the side preference, compared to 81% in an identical real-world study (Moussaïd et al., 2009, 2016; Figure 3). This suggest that participants in VR can consider other avatars as “real” people and expect them to follow similar social norms.

#### Simple evacuation

The second validation experiment focused on evacuation dynamics. Previous research has demonstrated that the outflow during an evacuation of a group of people increased linearly with the width of the room doorway (Kretz et al., 2006; Liddle et al., 2009; Seyfried et al., 2009; Daamen and Hoogendoorn, 2010).

**Figure 4:**
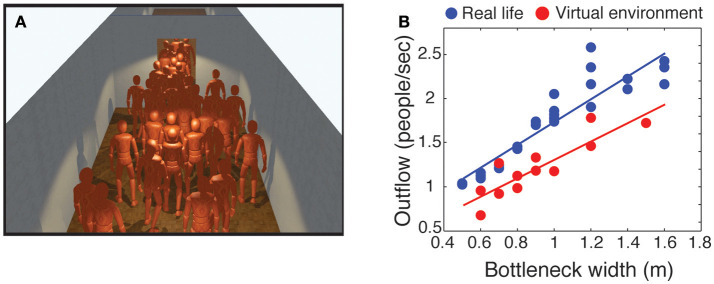
Simple evacuation. **(A)** Illustration of an experiment conducted in a virtual environment in which 36 participants were asked to evacuate a room through a doorway. **(B)** Outflow of people through bottlenecks of varying width measured in the virtual environment (red dots), replicating a real-world experiment (blue dots).

One of these evacuation experiments has been replicated in desktop VR (Moussaïd et al., 2016). A total of 36 participants were immersed simultaneously in a large virtual room and instructed to evacuate through a doorway of varying width (Kretz et al., 2006). Consistent with real-world findings, the outflow of pedestrians increased linearly with the bottleneck width (Figure 4). However, compared to a larger body of real-world datasets, the outflow of participants was smaller in the virtual environment. This difference can be attributed to micro-navigation factors such as differences in walking speed, acceleration, and/or shoulder movements.

### Emergency evacuations

Multi-user virtual environments also offer the advantage of enabling the investigation of difficult (if not impossible) scenarios. For example, the collective behavior that occurs during emergency situations (e.g., evacuating a burning building) can be challenging to study in the real world because of ethical and safety reasons (Schadschneider et al., 2011). Recently, emergency evacuations were investigated in virtual settings. Large groups of participants were instructed to evacuate a virtual building with four possible exits, only one which was not blocked by fire. For each trial, the location of the correct exit was randomly chosen, and only a randomly selected subset of participants were told which exit was correct. We compared collective behaviors between non-emergency and stressful emergency conditions. In the study, the two conditions differed by three factors. Specifically, in the stressful emergency condition, there was a short time limit imposed, participants were penalized for not finding the correct exit, and the environment contained stressful elements such as red blinking lights and a siren. In contrast, in the non-emergency condition, no time limit was imposed, participants were rewarded for finding the correct exit, and the environment lacked blinking lights and sirens.

**Figure 5:**
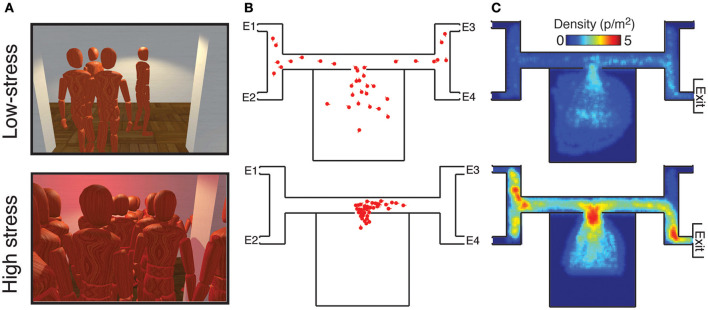
Emergency evacuations from the multi-user virtual environment. **(A)** Snapshots of the environment as perceived by a participant in non-emergency (top) and emergency conditions (bottom). **(B)** Bird's-eye view of the non-emergency (top) and emergency conditions (bottom). Each red dot represents the position of a participant in the virtual building a few seconds after the trial started. **(C)** Maximum density levels measured across the environment.

The results revealed significant differences between the two conditions (Figure 5). While participants searched for the exit in a slow and orderly manner in the non-emergency condition, mass herdings and severe crowding occurred in the emergency condition. In particular, in the non-emergency condition, participants tended to stay reasonably safe distances from one another in order to avoid a monetary penalty for colliding with each other. In contrast, a high number of collisions occurred in the high-stress condition, despite having the same collision penalty. Density levels remained lower than 2 people per m^2^ in the non-emergency condition, as typically observed in everyday congested zones (Still, 2000). Under high stress, the density level reached values up to 5 people per m^2^. This value is close to the critical threshold of crowd turbulence, a deadly collective phenomenon (Helbing et al., 2007). Another collective pattern that emerged in the emergency condition was herding. While participants in the non-emergency condition tended to choose a random branch at each intersection, the majority of participants herded in the same direction in the emergency condition, which amplified the crowding pattern.

The development of multi-user virtual environments for conducting crowd experiments is promising but still at the early stages. Additional validation experiments should be conducted. In addition, there are necessary improvements with respect to simulating social and physical interactions between avatars during navigation. For example, social interactions may be impacted by appearance and behavioral realism of other avatars in the virtual environment (e.g., gait; Narang et al., 2017b). These aspects of realism in VR can be improved using new methods for generating avatar movement based on the recordings of real people (Narang et al., 2017b). Empirical research has also demonstrated that the match between appearance and behavioral realism is critical for recognizing one's own movement (Narang et al., 2017a) and co-presence (Bailenson et al., 2005). With respect to crowds, Prazak and O'Sullivan (Pražák and O'Sullivan, 2011) suggest that the crowd's perceived realism depends on the number of animations particular to individual avatars.

Additional challenges for multi-user VR include the lack of haptic feedback and sound rendering, material constraints associated with equipping multiple participants with individual displays (e.g., HMDs) and controls, and sufficient training with these controls. Previous research has suggested the benefits (e.g., improved immersion) of haptic feedback using haptic garments (Ryu and Kim, 2004), vibrating actuators (Louison et al., 2017), and quadcopters (among others; see Knierim et al., 1998). Similarly, the rendering of spatialized sounds may complement visual feedback by providing temporal information that can improve presence (see Serafin et al., 2015 for a review). However, both haptic feedback and sound rendering require additional computing power and impose material constraints. For example, equipping 36 participants with HMDs, haptic garments, and spatialized sounds would be prohibitively expensive in terms of finances and computational resources. These constraints also require participants in multi-user VR to use simple controls such as a joystick. Training with these controls is critical given that participants may need to negotiate both static (e.g., walls) and dynamic (e.g., other avatars) obstacles (Grübel et al., 2017).

### Alternative approaches to multi-user experiments

Other approaches have been used to study crowd behaviors in VR. Compared to the above examples, these approaches are not presented from a first-person perspective and implement a less realistic graphical environment.

One of the first attempts to study evacuations in multi-user virtual environments was conducted within the popular massive multiplayer online game “Second Life.” There, users can create an avatar, explore a large virtual environment, and interact with other users' avatars. Whereas the primary purpose of Second Life is entertainment, researchers have used it to conduct behavioral experiments (Molka-Danielsen and Chabada, 2010; Normoyle et al., 2012). For these experiments, participants were recruited among existing users of Second Life with announcements posted in the virtual world. Participants met in a virtual building and then were asked to evacuate because of a virtual fire. The experimenters were able to characterize numerous aspects of emergency evacuations (e.g., exit choice, knowledge about the building plan), but this type of experimental setup offers little experimental control. Nevertheless, using an existing virtual world already populated with thousands of users could potentially allow the development of very large-scale experiments (i.e., with more than 36 participants). In addition, other massive multiplayer online platforms may allow for a combination of both larger crowds and experimental control to study phenomena such as crowd disasters.

Other simpler approaches for conducting crowd experiments in virtual environments have also been developed. For example, Bode and Codling have studied various aspects of evacuation dynamics by having participants control the movement of a dot with a computer mouse through a two-dimensional environment from a top-down perspective (Bode and Codling, 2013; Bode et al., 2014, 2015). The authors managed to highlight some important aspects of participant behavior during evacuations, such as the impact of congestions, static signs, social cues, and memorized information on routing and exit choice dynamics. Although these experiments were designed for a single participant interacting with simulated agents, adapting this approach to multiple simultaneous users should only present minor technical challenges.

Similarly, the HoneyComb paradigm has a multi-player design in which each participant controls a dot on a two-dimensional playfield (Boos et al., 2017). Using their mouse, groups of participants can navigate simultaneously in a shared environment. Every individual can see the position and the movement of the those who are located within a particular perceptual radius. In such a way, researchers investigated a series of fundamental questions related to the role of leadership (Boos et al., 2014), spatial attraction (Belz et al., 2013), and competition (Boos et al., 2015) on collective flocking patterns.

## Discussion

Conventional methods of crowd monitoring are difficult to implement for tracking large crowds, and experimental approaches often face organizational and ethical challenges. Owing to recent technological developments, novel methods of crowd monitoring (i.e., virtual sensing) and crowd experimentation (i.e., multi-user virtual reality) have emerged and constitute promising complementary options for crowd researchers.

### Virtual sensing

Most pedestrians carry a connected device (e.g., a smartphone) that continuously emits radio-frequency signals. Whereas, the physical locations of individuals are often difficult to establish using video recordings, these locations can be inferred by detecting and tracking the virtual traces left by their devices. Crowd monitoring techniques have rapidly evolved from manual counting to computer-based video analyses. Researchers can now transition toward virtual sensing techniques. However, two major challenges for this approach are to access a sufficiently large proportion of these signals and to estimate their locations as accurately as possible.

Toward this end, two methods have been developed. The first method consists of distributing a dedicated application to a large sample of users. This application can continuously record users' positions and send these positions to a central server. The second method consists of monitoring the Wi-Fi signals emitted by devices using dedicated sensors installed in the area of interest. Both methods are able to accurately represent the crowd's movement and density. However, both methods also require a considerable amount of effort to set up. Deploying an application requires a marketing effort to distribute as broadly as possible and convince people to install and activate it. Remarkable progress has been made in that regard between the two past deployments (at the Lord Mayor Show in London and the 2013 Züri Fäscht in Zürich) of a virtual sensing system, for which the number of participants has increased from 828 users to 28,000 users. In particular, the authors of these studies noticed that the application should offer a variety of services to the users, explicitly communicate about what usage is made with the collected data, and make use of social networks and social recommandation tools.

In contrast, monitoring Wi-Fi signals does not require the explicit cooperation of the individuals. However, dedicated signal sensors must be installed in the area of interest and may require permission from the event organizers. In addition, the sensors must be positioned as much above the crowd as possible in order to avoid signal interruptions and obstructions. Recently, innovations in animal tracking have demonstrated the advantages of using drones to collect video and GPS data on the movement of wild baboons (Strandburg-Peshkin et al., 2017). Similarly, one could imagine embedding radio-frequency sensors in drones flying above the crowd, which could minimize signal interruptions and convert the sensor into a mobile installation.

Another branch of virtual sensing employs the traces left by interactions between people on the phone or the Internet. To date, such an approach has been used to collect macroscopic data such as unemployment levels, disease prevalence, and consumers behavior based on Internet search queries (Ginsberg et al., 2008; Goel et al., 2010). People's positions in space can also be inferred from their activity patterns. For example, Gonzalez and colleagues used the data from a mobile phone carrier containing the date, time, and coordinates of the phone towers routing the phone calls of ~6 million users (González et al., 2008). The movements of each user was then inferred by tracking the locations of the phone towers routing the communications despite low spatial resolution (~3 km^2^) and restrictions regarding data accessibility. Nevertheless, it has been shown that the spatial density of phone communications correlated with the volume of geolocalized tweets recorded over the same period on Twitter (Botta et al., 2015). In other words, the number of tweets and the place where they were produced—free and easily accessible data—can serve as a proxy to estimate the density of people in a certain area of interest.

In general, virtual sensing approaches remain less accurate than conventional video-based tracking methods. The positioning of the individuals is, at best, estimated within a few meters of uncertainty. This challenges the extraction of individual-level mechanisms underlying the crowd dynamics. However, virtual sensing has a larger spatial and temporal reach, potentially covering an entire city during unlimited time periods. As such, both methods complement each other well and should eventually constitute different options in the crowd researcher's toolbox.

### Virtual reality

While virtual sensing allows for the observation of natural crowds, multi-user virtual reality provides more control over experimental conditions and the ability to draw causal inferences. This approach builds on single-user virtual reality by allowing for the study of simultaneously immersed users. These multi-user virtual environments have several other advantages.

First, virtual environments are easy to manipulate. Researchers can conduct experiments in virtual buildings, streets, stadiums, or large vehicles such as planes and boats of different typologies and sizes. Unlike real-world experiments that rely on existing physical infrastructures, virtual designs can modify existing environments or create new ones. For example, the side preference experiment described above was conducted both in the real world and in the virtual environment. In the real world, 144 replications of the experiment were collected during several days. In the virtual world, 561 replications of the same experiment were collected in <15 min. Regarding the creation of new environments or situations, experiments can be conducted to address questions that were previously unapproachable because of safety or ethical issues. For example, they enable the systematic investigation of crowd behavior under stressful and dangerous conditions with real human participants.

Second, multi-user virtual environments allow for greater experimental control. For example, experimental variables such as light level, walking speed, and body size may be manipulated in a way that is not possible in real-world settings. Experiments could also modify real participant behavior to create artificial agents and induce the propagation of certain behaviors through the crowd.

Third, experiments in multi-user virtual environments allows the collection of a large variety of measurement variables with high precision. Participants' positions, speeds, and body and head orientations can be easily captured at high resolution and with minimal measurement errors. In addition, other types of behavior could also be measured such as properties of participants' gaze (using eye trackers) and their physiological states (using electrocardiograms or skin conductance sensors).

While some researchers have studied crowd behavior online with mixed results, new technologies may allow for carefully controlled multi-user experiments in the near future. In such scenarios, participants could use their own computer setup and participate from home. It may be unrealistic to expect a large number of participants at their respective homes wearing HMDs for research purposes. However, desktop computers with mouse and keyboard setups may be sufficient for some experiments similar to those already conducted in the laboratory. These advancements suggest that massive online crowd experiments could be used for studying thousands of participants connected to an experimental server at a given moment of time. Previously, similar group experiments were conducted in the fields of social psychology and network science (Mason and Watts, 2012; Mao et al., 2016). In these experiments, up to a 100 of online participants were tested simultaneously, This approach could be further facilitated by the existence of crowdsourcing platforms for recruiting participants such as Amazon Mechanical Turk or Prolific Academic (Mason and Suri, 2012).

Despite these advantages, multi-user virtual reality cannot be considered as a replacement for conventional real-world experiments. It offers some advantages, like a greater control on external variables, the ease of designing environments, and the potential for exploring dangerous situations, but also has drawbacks. For example, the feeling of body contacts in high density situations is difficult to communicate realistically. Similarly, there exist numerous micro-navigation differences that prevent participants from modulating their speed and acceleration as they would in real life.

## Conclusion and perspectives

In this review, we have described two technological innovations that can offer promising new perspectives for crowd researchers. While live monitoring techniques can facilitate data collection for field studies, multi-user virtual reality offers new opportunities for conducting experiments with greater flexibility and control. Similar developments are also taking place for the study of other self-organized social systems. Animal tracking methods are currently undergoing major changes with the development of high-accuracy GPS methods (e.g., Nagy et al., 2010; Strandburg-Peshkin et al., 2015, 2017). At the same time, virtual reality is emerging as a powerful tool for studying social interactions among fish and understanding the resulting collective behaviors of the school (Ioannou et al., 2012; Stowers et al., 2017). The parallel development of virtual sensing and virtual reality across different social systems confirms the important role that these two methods might play for the study of the self-organized crowd phenomena in the future.

## Author contributions

MM conceived and structured the review. MM, VS, MK, and TT wrote the paper.

### Conflict of interest statement

The authors declare that the research was conducted in the absence of any commercial or financial relationships that could be construed as a potential conflict of interest.
